# Training the next generation of physician researchers – Vanderbilt Medical Scholars Program

**DOI:** 10.1186/s12909-017-1103-0

**Published:** 2018-01-04

**Authors:** Abigail M. Brown, Teresa M. Chipps, Tebeb Gebretsadik, Lorraine B. Ware, Jessica Y. Islam, Luke R. Finck, Joey Barnett, Tina V. Hartert

**Affiliations:** 1 Outcomes Research, Biomedical Research Education & Training, Clinical & Translational Scientist Development, Vanderbilt University, Nashville, Tennessee USA; 2 Center for Asthma & Environmental Sciences Research, Vanderbilt Environmental Health Science Scholars Program (NIEHS K12), Vanderbilt Medical Scholars Program, Vanderbilt University School of Medicine, Nashville, Tennessee USA; 3 Department of Biostatistics, Vanderbilt University Medical Center, Nashville, Tennessee USA; 4 Vanderbilt Medical Scholars Program, Division of Allergy, Pulmonary and Critical Care Medicine, Department of Medicine, Vanderbilt University School of Medicine, Nashville, Tennessee USA; 5 Vanderbilt Medical Scholars Program, Division of Allergy, Pulmonary and Critical Care Medicine, Department of Pathology, Microbiology and Immunology, Vanderbilt University School of Medicine, Nashville, Tennessee USA; 6 Department of Epidemiology, University North Carolina, Chapel Hill Gillings School of Global Public Health, Chapel Hill, North Carolina USA; 7 Center for Asthma & Environmental Sciences Research at Vanderbilt University Medical Center, Nashville, Tennessee USA; 8 Office of Medical Student Research, Health Sciences Education, Vanderbilt University School of Medicine, 312 Light Hall, Nashville, TN 37232-0301 USA; 9 Physician Researcher Development, Office for Medical Student Research, Vanderbilt University School of Medicine, Nashville, Tennessee USA; 10 Translational Science, Center for Asthma Research, Department of Medicine, Division of Allergy, Pulmonary and Critical Care Medicine, Vanderbilt University School of Medicine, Nashville, Tennessee USA

**Keywords:** Undergraduate medical education, Research, Training, Physician researchers

## Abstract

**Background:**

As highlighted in recent reports published by the Physician-Scientist Workforce Working Group at the National Institutes of Health, the percentage of physicians conducting research has declined over the past decade. Various programs have been put in place to support and develop current medical student interest in research to alleviate this shortage, including The Vanderbilt University School of Medicine Medical Scholars Program (MSP). This report outlines the long-term program goals and short-term outcomes on career development of MSP alumni, to shed light on the effectiveness of research training programs during undergraduate medical training to inform similar programs in the United States.

**Methods:**

MSP alumni were asked to complete an extensive survey assessing demographics, accomplishments, career progress, future career plans, and MSP program evaluation.

**Results:**

Fifty-five (81%) MSP alumni responded, among whom 12 had completed all clinical training. The demographics of MSP alumni survey respondents are similar to those of all Vanderbilt medical students and medical students at all other Association of American Medical College (AAMC) medical schools. MSP alumni published a mean of 1.9 peer-reviewed manuscripts (95% CI:1.2, 2.5), and 51% presented at national meetings. Fifty-eight percent of respondents reported that MSP participation either changed their career goals or helped to confirm or refine their career goals.

**Conclusions:**

Results suggest that the MSP program both prepares students for careers in academic medicine and influences their career choices at an early juncture in their training. A longer follow-up period is needed to fully evaluate the long-term outcomes of some participants.

**Electronic supplementary material:**

The online version of this article (10.1186/s12909-017-1103-0) contains supplementary material, which is available to authorized users.

## Background

In the field of biomedical research, physician scientists bring a unique clinically driven perspective and play a vital role in the advancement of health care practice through the translation of new discoveries into clinical care. “Physician-scientists are individuals with an MD degree who perform medical research as their primary professional activity.” [1] Such physicians are able to develop clinically relevant questions pertinent to the improvement in quality of care for patients [1]. However, the declining trend in physician-scientists is well documented [2–5] and is attributed to several factors, including: structural failures in the development pipeline [6], economic and intellectual challenges [7], growing debt of medical students, increased length in post-doctoral training required for a successful research career and inherent instability in a National Institutes of Health (NIH) funded research career [8].

In light of this documented decline, several programs were created to support the development of the physician-scientist career path. Such initiatives include several NIH sponsored career development awards (K08, K23, K24 and K30) and the NIH Loan Repayment Program (LRP) [9]. The Howard Hughes Medical Institute (HHMI) offers the Medical Fellows and the Cloister programs, which are targeted at attracting medical students toward careers in research. Outcomes from these programs include 16% of medical fellows and 20% of Cloister graduates engaging in academic careers and 24% of medical fellows and 21% of Cloister receiving NIH awards [10]. Though these results may be discouraging, there is evidence to suggest that exposure to research experiences may increase the number of physician-scientists [11–14] and is associated with improved productivity and more informed trainees and has shown to result in an increased interest toward research [16]. A recent meta-analysis suggests medical students whom participate in research are over three times as likely to report interest in research engagement in their future careers, are six times as like to purse ‘academic careers’ as compared to their “untreated” peers and are twice as likely to academically outperform their peers [15].

Several institutions, including Vanderbilt University School of Medicine (VUSM), implemented institutionally funded undergraduate medical education research programs to help combat this nationally growing concern [16–21]. Research experiences, rooted in the practice of evidence-based medicine, are commonly recognized as an important component of physician training resulting in the development of curricula globally (e.g., in the United Kingdom [22], Canada [23], Turkey [24], and the United States [25]). The impact of early research training, particularly during undergraduate medical training, has been proven to be significant in career development [8, 25–28]. VUSM Medical Scholars Program (MSP) was created in 1998 to encourage and provide the means for medical students in Nashville, TN to spend an additional funded in-depth research year in medical school.

The VUSM Medical Scholars Program (MSP) is a competitive, one-year, mentored research experience available to medical students at both VUSM and Meharry Medical College, the two medical schools in Nashville, TN. The MSP aims to provide an early research experience, foster an interest and build skills in research that may lead to the pursuit of careers in academic medicine. This report describes initial efforts, following surveyed alumni to assess their careers after program completion in order to determine if the program has achieved these initial goals.

## Methods

### Survey data

MSP alumni were asked to complete an extensive survey beginning in December 2008 (see supplemental on-line content, Additional file 1). This study was approved by the Vanderbilt University School of Medicine Internal Review Board (IRB). Former MSP participants were informed that they were giving their implied consent by completing the surveys.

### Statistical methods

We performed a cross-sectional analysis of the MSP alumni survey responses (see Additional file 2). Variables were described as frequencies and proportions and ordinal or continuous scales as mean and standard deviation or median and interquartile range [IQR]. Where appropriate, aggregate alumni responses and their corresponding 95% confidence intervals (CIs) were calculated for comparison to the national or Vanderbilt-specific 2010 AAMC Graduation Questionnaire data [29].

### Program outcome assessment variables

The main variables used to assess factors associated with pursuit of a career in academic medicine and the development of independent scientific careers were demographic (gender, race/ethnicity, age), scholastic achievement (MCAT, undergraduate GPA scores, academic honors, such as AOA), specialty selection, and academic debt. Individual accomplishments attributable to the MSP program were assessed by published manuscripts and national meeting presentations that covered research completed during the MSP year. Career goals and current career type were additionally assessed and compared with both Vanderbilt medical school data and AAMC graduation report data. Analyses were conducted using R version 2.15.3 ((www.r-project.org, R Development Core Team (2009) [30]; Vienna, Austria)) and GraphPad Prism version 5.04 for Windows, (GraphPad Software, San Diego California USA, www.graphpad.com).

## Results

### Respondent demographics

In total, 55 out of 68 (81% response rate) alumni completed part one of the alumni survey. The demographics of MSP alumni survey respondents are similar to those of all Vanderbilt medical students and medical students at all other AAMC medical schools. Table 1 summarizes the self-reported demographics for MSP alumni; 2010 AAMC Medical School Graduation Questionnaire final report for Vanderbilt University School of Medicine (Vanderbilt 2006–2010) and all AAMC schools (All Schools 2010) are included for comparison.

**Table 1:** MSP survey respondent demographics in comparison with all Vanderbilt Medical School graduates and AAMC data on all medical school graduates

Variable	MSP Alumni Survey Respondents^a^		All MSP Alumni		All Vanderbilt Medical Students 2006-2010^d^		All Medical Schools 2010	
	*N* = 55		*N* = 80		*N* = 295		*N* = 13,922	
Gender								
Female	28	(51%)	36	(45%)	162	(56%)	6876	(49%)
Male	27	(49%)	44	(55%)	133	(45%)	7044	(50%)
Race N (%)								
Asian	11	(20%)			40	(17%)	3202	(23%)
Black/African American	2	(4%)			19	(8%)	988	(7%)
White/Caucasian	42	(76%)			189	(77%)	10,066	(72%)
Other, including multiple races	1	(2%)					251	(1.8%)
Ethnicity, Hispanic	2	(4%)					1002	(7.2)
Age in years at time of survey (mean ± s.d.)	31.9 ± 3.3							
Alpha Omega Alpha (AOA) Honor Medical Society member, N (%)	10	18%						
Undergraduate GPA (mean ± s.d.)	3.8 ± 0.2				3.75 ± 0.20			
Total MCAT score (mean ± s.d.)	31.3 ± 9.9				33.4 ± 3.28			
Current position at time of survey, N (%)								
Medical student	7	15%						
Intern	1	2%						
Resident	25	45%						
Clinical fellow	10	18%						
Postdoctoral research fellowship	1	2%						
Postgraduate clinical degree program only	1	2%						
Completed all clinical training	10	18%						
Number of hours working per week at time of survey, mean ± s.d.	61 ± 15							
First residency^b^, N (%)								
Internal medicine	17	(31%)			23	11%	1706	15%
Surgery^c^	9	(16%)			44	(21%)	1494	(17%)
Combined Medicine-Pediatrics	3	(7%)						
Pediatrics	4	(7%)			12	(6%)	1070	(10%)
All other	22	(38%)						
Amount of academic debt, N (%)								
$0-$99,000	24	(43%)			61%§		33%§	
$100,000-199,000	23	(42%)			20%		41%	
≥ $200,000	8	(15%)			20%		26%	

^a^MSP Alumni Survey was conducted in 2008-2009

^b^For individuals doing a subspecialty fellowship, the choice of specialty may not be the same as the first residency

^c^Includes General Surgery and Surgery (Other: ENT, orthopedics, plastic surgery, urologic surgery, etc.)

^d^All Vanderbilt Medical Students 2006-2010 and all medical school data: Medical school graduation questionnaire, individual school report

### Postgraduate training

At the time of the survey, 47 MSP alumni (85%) had completed medical school. Thirty-three percent of MSP alumni obtained additional degrees. The details of post-graduate training are summarized in Table 1 and in the supplemental online content.

### Accomplishments during medical scholars program

MSP alumni reported publishing a mean of 1.9 peer-reviewed manuscripts (95% CI:1.4, 2.4) and 0.9 first-author, peer reviewed manuscripts (95% CI: 0.6, 1.1) that directly arose from their MSP research year. A majority of MSP alumni (67%) published at least one peer-reviewed manuscript, and 49% published at least one first-author, peer-reviewed manuscript. In a recent meta-analysis, an average of 30% (95% CI 0.19–0.44) of research performed by medical students resulted in a peer-reviewed journal publication [15]. Fifty-one percent of MSP alumni have also presented their MSP research results at regional, national, or international academic meetings. Similar programs report that approximately 50% of students deliver a presentation at an extramural meeting as a result of participating in similar experiences [15]. Funding for meeting attendance was not implemented with the initiation of the MSP, thus early program participants would not have had this opportunity. MSP alumni reported a mean of 2 presentations (95% CI: 1.4, 2.6) at national or international meetings.

### Current careers of MSP alumni

**Fig. 1:**
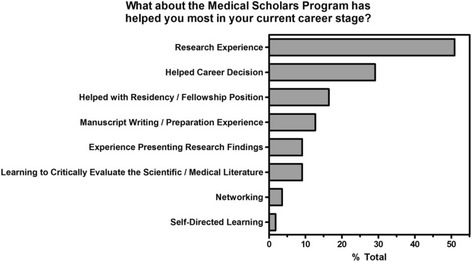
Medical Scholars Program features that participants stated helped them most in their current career stage. Alumni were able to indicate more than one reason

At the time of the survey, 55 MSP alumni were in a variety of different career stages: medical student (7), intern or resident (26), research or clinical fellow (11), and 10 had completed all clinical training (Table 1). In response to the question, “What about the Medical Scholars Program has most helped you in your current career stage?” alumni gave a variety of free-text answers. These answers are grouped into eight different categories (Fig. 1) with “Research Experience” and “Helped Career Decision” the most frequent responses.

While the 55 alumni were in a variety of different career stages at the time they took the survey, 12 (21.8%) had at least half of their salary supported through grants or other external funding sources. In addition, 25 alumni (45.5%) have written or participated in writing a grant to obtain funding for all or part of their salary and/or a project. Eleven alumni (20%) currently serve (or have served) as Principal Investigator on one or more grants.

### Long-term career goals

Among the 55 MSP alumni who reported their long-term career focus, the majority of responses included: research and clinical care (35%), teaching and clinical care (11%), global health (4%), research and teaching (4%), and research, teaching and clinical care (2%) (Table 2). Forty percent reported a desire to be significantly involved with research during their future careers. A majority (55%) of MSP alumni indicated an interest in an academic faculty position (Table 2). Data on careers from the 2010 AAMC Medical School Graduation Questionnaire final report for Vanderbilt University School of Medicine (Vanderbilt 2010) and all AAMC schools (All Schools 2010) are included for comparison (Table 2). Notably, the percentage of MSP alumni who chose a career as a non-academic clinician is similar to all other Vanderbilt students and is less than half of the All Schools 2010 cohort (Table 2). A larger proportion of students indicated interest in significant future involvement in research (40%) compared with all Vanderbilt (31%) and AAMC (16%) students (Table 2). In contrast, a larger proportion of MSP participants indicated they were undecided regarding their future career paths (20%) compared with all Vanderbilt (11%) and other AAMC schools (17%).

**Table 2:** Long-term career goals and desire for future involvement in research. The type of academic faculty position is shown in detail for only MSP alumni

Long-term Career Goals	MSP Alumni (*n* = 55)		Vanderbilt 2010^a^ (*n* = 71)	All Schools 2010^a^(*n* = 13,144)
	N	%	%	%
Academic faculty	30	55%	70%	43%
Research and teaching	2	4%		
Research and clinical care	19	35%		
Teaching and clinical care	6	11%		
Research, teaching, and clinical care	1	2%		
Global health emphasis	2	4%		
Clinician (non-academic)	8	15%	17%	35%
Medical/Healthcare administration	3	5%	0%	0%
Government agency	1	2%	0%	2%
Other	2	4%	1%	2%
Undecided	11	20%	11%	17%
Future interest in involvement in research				
Exclusively involved	0	0%	1%	0%
Significantly involved	22	40%	31%	16%
Somewhat involved	18	33%	48%	45%
Involved in a limited way	14	26%	31%	31%
Not involved	1	2%	3%	8%

^a^All Vanderbilt Medical Students 2006-2010 and all medical school data: Medical school graduation questionnaire, individual school report

When asked if participation in the MSP resulted in a change in career goals, students gave a variety of free-text responses that were grouped into three categories: MSP participation “changed career goal” (*n* = 20, 36%), MSP participation helped to “confirm or refine career goal” (*n* = 12, 22%), and MSP participation had “no impact on career goal” (*n* = 23, 42%). Of the 20 alumni who indicated that the MSP changed their career goals as a result of participating in the program, 11 (55%) have now chosen to pursue careers as academic faculty members, while the remainder either want to pursue careers as full-time clinicians (*n* = 6) or were undecided (*n* = 3). Of the 12 alumni that said the MSP helped to confirm or refine their career goals, 10 (83%) have chosen to pursue careers as academic faculty members, while the remainder want to work in a government / non-profit agency or on global health policy (*n* = 2). Of the 14 alumni who reported a change in their career goals which was not due to their participation in the MSP, four (29%) have now chosen to pursue careers as academic faculty members, while the remainder either want to pursue careers as full-time clinicians (*n* = 2), medical administration (*n* = 3), government/non-profit agency (*n* = 1), or were undecided (*n* = 4).

### Alumni recommendations

**Fig. 2:**
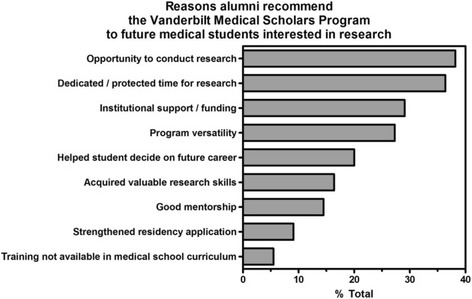
Reasons alumni recommend the Vanderbilt Medical Scholars Program to future medical students interested in research

**Fig. 3:**
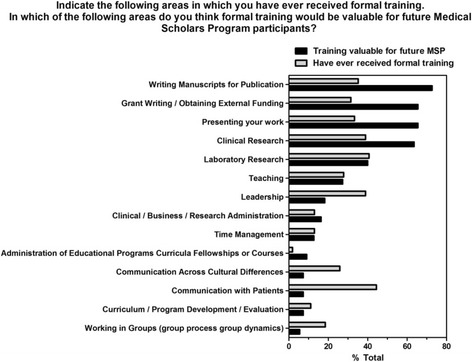
Areas that alumni think formal training would be valuable for future MSP participants. Alumni indicated areas they had ever received formal training (grey) and areas they think would be valuable for future MSP participants (black). Survey respondents were able to choose multiple areas in their answer. The X axis represents % total responding “yes”

Vanderbilt MSP alumni were asked to provide reasons why they would recommend the program to future medical students interested in research. The majority of responses highlight the opportunity to conduct research and dedicated/protected time to conduct research (Fig. 2). MSP alumni were also asked to identify the most valuable aspects of the program for future participants. More than 60% of MSP alumni highlighted the importance of training related to scholarly communication (manuscript development, grant writing and presenting work) and clinical research (Fig. 3).

### Academic debt

Although our sample size is small, those who pursue the MSP are less represented at the highest levels of debt (≥ $200,000: 15%) compared to both their peers at Vanderbilt (≥ $200,000: 20%) and all other medical school students (≥ $200,000: 25%) (Table 1). More Vanderbilt MSP alumni reported a high amount of academic ($100,000 - ≥ $200,000: 57%) debt compared to their peers at Vanderbilt (($100,000 - ≥ $200,000: 40%). Among those who pursued careers as academic faculty, 52% (*N* = 14/27) of MSP alumni had academic debt greater than $100,000, and 48% (*N* = 13/27) of MSP alumni had academic debt less than $100,000.

## Discussion

In 1998, Vanderbilt invested in creating a medical student research program, the Vanderbilt Medical Scholars Program (MSP), to train a select group of medical students in an intensive, additional-year, research training experience. The Vanderbilt MSP builds on the existing strengths of our institution to meet the national needs for clinically trained biomedical investigators. The fundamental questions of how we attract medical students to a career as a physician-scientist and what attributes and/or experiences contribute to selecting this path are of germane interest to us. The current report focuses on the initial efforts to survey the career paths of alumni who have completed their training since the inception of this program.

### A worthwhile investment?

Analysis of these results suggests that this type of intensive training experience can help to prepare students to pursue careers in academic medicine, as well as help them decide on a career (Fig. 1). Program participation was most valued by students for the opportunity to perform research and aid in their career decisions (Fig. 1). Even if participants chose not to pursue a career in academic medicine, the MSP experience can assist in further critical appraisal and independent learning skills, which are important for any physician leader [12, 31].

Although the cost of investing in this type of program may be daunting for institutions, the data on productivity and desire for significant future involvement in research for MSP participants suggests that such an investment may be worthwhile. Of particular interest to the authors are those areas that former MSP thought were especially valuable skills gained during the course of MSP participation including writing, grant writing, presentations and clinical research (Fig. 1). Such areas of training should be incorporated into formal medical training for all, and be included as targets towards the development of global medical student research education programs.

Fifty-five percent of MSP alumni (*n* = 13) indicated an interest in academic faculty positions; a value higher than all other schools (*n* = 144, 43%), but less than that of all Vanderbilt Medical Students (*n* = 71, 70%). However, the MSP data reveals that in addition to 55% (*n* = 30) who now profess strong interest in academic careers, 40% (*n* = 22) also foresee significant involvement in research versus 31% for all Vanderbilt Medical Students and 16% for all AAMC students. In addition, 11% (*n* = 6) indicate interest in administrative, government agency, or other careers compared to 1% (*n* = 1) of all Vanderbilt Medical Students who state these areas of interests. Administrative and government careers often involve research and leadership components that make these careers a desirable outcome for MSP graduates. In addition, MSP graduates also report a higher rate of undecided for career choice (*n* = 11, 20% versus *n* = 8, 11% for all VMS students). Fifteen percent (*n* = 8) of MSP graduates pursue non-academic clinical careers compared to 17% (*n* = 12) of all Vanderbilt Medical School graduates. Taken together, these data suggest that the MSP experience foster the consideration and evaluation of wider career choices and opportunities than that seen in nonparticipants. Though these findings involve a small cohort, they support previous studies suggesting that exposure to such experiences may increase the number of physician-scientists [5, 11–15]. Further long term evaluation of the career paths of MSP graduates will clarify the impact of the program on not only academic careers, but other research and leadership career pathways including administration and government service.

### Limitations & future research

This current retrospective study is descriptive in nature and is not without limitations. A true comparator group was unavailable as students who do not participate in the MSP were not required to complete a research experience. Future studies may be strengthened by including comparator groups (e.g., looking at the publication and/or presentation rates for those who did not participate in the program). The study is limited by the response rates and possible bias of participants. Tracking and maintaining an accurate alumni database is easier with a small cohort, but does require regular maintenance and updating. In addition, access to certain financial data is currently limited or unavailable.

It is important to highlight that applications of a similar program to systems outside of the United States (e.g., European, Common Wealth) may be limited due to institutional and structural differences of these systems. Similarly, variations exist on how outcome data is evaluated and reported among differing programs. Programs may require outcomes such as presentations or the production of a publishable manuscripts, while others may have different criteria and/or goals [32]. Therefore, associations between participation and productivity does not prove causality.

Data from this initial report indicates that involvement may have helped participants decide not to pursue research as a career as they were able to better understand what is involved and the commitment associated with research, which could avoid additional periods of training and additional fiscal resource utilization. While the relatively short follow-up period for some participants does not permit us to evaluate the long-term success of all participants in their pursuits of careers in academic medicine, these early results suggest that such a program both prepares students for careers in academic medicine and influences their career choices at an earlier juncture in their training.

The selection, development and support of physician scientists is essential for the productivity, and indeed survival, of academic medicine. Identification of the optimal interventions that foster successful navigation of the physician-scientist pathway is complicated by both the changing academic environment and the challenges inherent in performing and applying studies that address long-term outcomes. This initial effort begins this tracking process and we plan to continue to document and report the outcomes of the program.

## Conclusion

Since the inception of the Medical Scholars Program (MSP) in 2012, the experience and early results from this program have helped to shape a new undergraduate medical education research program for all students at Vanderbilt University School of Medicine. Similarly, the experiences and outcomes from this larger group of students will help us learn how to better prepare students to choose future careers, how to best educate undergraduate medical students about biomedical research through an experiential program, and how to develop their critical thinking, creativity, leadership, moral and civic capacities to the fullest. This program aims to train physicians with a rich awareness of not only the clinical realm, but also research methods, the critical evaluation of research, and the understanding of the contribution of research to our clinical evidence base, so that they may be physicians who can effectively serve and lead in their chosen professions.

## Additional files

Additional file 1: Supplemental On-line Content. (DOCX 17 kb)

Additional file 2: Survey. (PDF 770 kb)

## Abbreviations

AAMC: Association of American Medical College
CI: Confidence Intervals
HHMI: Howard Hughes Medical Institute
IRB: Internal Review Board
LRP: Loan Repayment Program
MSP: Medical Scholars Program
NIH: National Institutes of Health
VUSM: Vanderbilt University School of Medicine
