# Supplementary material for: Training the next generation of physician researchers – Vanderbilt Medical Scholars Program
Source: BMC Med Educ. 2018 Jan 4;18:5. doi: 10.1186/s12909-017-1103-0 (PMC5753449; doi:10.1186/s12909-017-1103-0)
Supplement: Supplementary file 1 — Supplemental On-line Content. (DOCX 17 kb) [file 12909_2017_1103_MOESM1_ESM.docx]

**Supplemental On-line Content**

**Training the Next Generation of Physician Researchers – Vanderbilt Medical Scholars Program**

Authors: Abigail M. Brown PhD1, Teresa M. Chipps BS2, Tebeb Gebretsadik MPH3, Lorraine B. Ware MD4, Jessica Y. Islam, MPH5, Luke R. Finck EdD MA6, Joey Barnett, PhD7, Tina V. Hartert MD MPH8

1. Program History
2. Program Governance & Oversight
3. Applicant Evaluation and Selection
4. Survey Methods
5. Post-graduate training
6. Alumni Evaluation and Recommendations
7. List of Grant Support of MSP Alumni
8. Survey instruments

# I. Program History

The MSP was formed in 1998 as an institutionally funded, one-year research program for medical students based on the belief that training in research methods is a critical part of medical education. Financial resources from the Vice Chancellor’s Office and the Office of the Dean funded the entirety of the MSP at its inception. At the present time, the Office of the Dean provides 73% and NIH provides

approximately 27% of the cost of the MSP. To our knowledge, Vanderbilt’s MSP is one of only a few internal, pre-doctoral fifth-year research programs supported by institutional funds30. Applicants to Vanderbilt’s MSP are encouraged to co-apply to similar national medical student training programs to which their research interest is aligned, including the Howard Hughes Research Program31, the Doris Duke Charitable Foundation32, and the National Institute of Diabetes and Digestive and Kidney Diseases (NIDDK)33, among others.

The MSP is overseen by a director, formerly Tina Hartert, MD, MPH, and currently Lorraine B. Ware, MD, has an Internal Advisory Board and is housed within the Vanderbilt University School of Medicine’s Office of Medical Student Research. Matching potential MSP students with research mentors is done on an individual basis. This ensures that during their year of research, scholars obtain a strong core education in research approaches and intensive training in scientific inquiry using a preceptor- oriented, project-based approach. Project selection and development occurs between the mentor and the applicant, and project requirements include an infrastructure to support the research. MSP participants are provided stipends that are commensurate with NIH guidelines, as well as funding to present their research at national meetings. During the MSP, medical students spend an additional year from their traditional four-year medical education to conduct research under the mentorship of faculty at Vanderbilt or at other U.S. or international academic institutions.

# III. Applicant Evaluation and Selection

Annual applications to the Vanderbilt Medical Scholars Program undergo a competitive selection process. To be selected to participate in the program, competitive applicants must (1) be in good standing at their respective medical schools, (2) demonstrate a recognized commitment to pursue research, (3) identify an appropriate research project, and (4) identify a mentor who will foster and support the applicant’s research project. Candidates submit applications that include a research plan, personal statement, and letters of recommendation. The applicant’s proposed mentor also submits an application that includes the mentor’s CV, a list of current funding, a list of current and previous trainees, and a mentoring plan for training the student. Applications are reviewed by the MSP Advisory Committee, which is comprised of senior research leaders in the medical center. Applications are ranked on an NIH scale, with equal weight given to the candidate, the mentor, and the feasibility and importance of the proposed project.

The survey consisted of two unlinked sections that contained up to 66 questions. Surveys were completed either online or via US mail, and respondents were not required to answer every question. The first section of the survey was anonymous and asked alumni questions about their demographics, productivity, career progress after completing the MSP, future career plans, and an overall MSP program evaluation. The second part of the survey was identifiable and asked alumni for specific contact information so they could receive a monetary gift card to compensate them for their time for survey completion. Surveys did not obtain any information that would allow part one and part two of the survey to be matched for any given individual.

# V. Post-graduate Training

At the time of the survey, 47 MSP alumni (85%) had completed medical school. The details of post-graduate training are summarized in Table 1. Of this group, 41 (87%) entered a clinical internship (16), a traditional clinical residency (21), or a fast-track clinical residency (4) immediately after finishing medical school. The remaining 13% entered the Medical Scholars Program (2), a postdoctoral research fellowship (3), or a Masters in Public Health program (1) immediately after completing medical school. Table 1 summarizes the four most common types of first residencies selected by MSP alumni: internal medicine, surgery, combined medicine-pediatrics, and pediatrics. Choice of specialty from the 2010 AAMC Medical School Graduation Questionnaire final report for Vanderbilt University School of Medicine (VU MD Students 2006-2010) and all AAMC schools (All Schools 2010) is included for comparison. Nine MSP alumni have served as Chief Resident. Following residency, 14 out of 20 (70%) eligible alumni chose to do a fellowship.

Thirty-three percent of MSP alumni obtained additional degrees. Six alumni earned concurrent degrees while in medical school [MA, MS or MSCI (n=2), MPH (n=2), PhD (n=1), JD (n=1)], and twelve following medical school [MA, MS, or MSCI (n=4), MPH (n=2), MBA (n=6)].

All respondents said that they would recommend the Medical Scholars Program to future medical students interested in research. Alumni were asked why they recommended the program, and their free- text responses were grouped into nine categories (Figure 2). The most frequent reasons given for recommending the MSP were: (a) opportunity to conduct research, (b) dedicated / protected time for research, (c) institutional support / funding, (d) sufficient versatility, and (e) helped student decide on a future career. MSP alumni were asked to indicate the specific career development areas they thought would be valuable training for future MSP participants and whether they had ever received formal training in these areas. These included: writing manuscripts for publication, grant writing / obtaining external funding, presentation skills, and clinical research (Figure 3). Less than 40% of alumni reported ever having received prior formal training in these four areas.

# Grant Support of MSP Alumni

Listed grant support of MSP Alumni include: private foundation career development award (e.g., Doris Duke, Burroughs-Wellcome), NIH K-series award, research grant from a non-NIH governmental agency, research grant from a private foundation (e.g. Doris Duke, Burroughs-Wellcome), NIH postdoctoral fellowship award (F32), and NIH training grant (T32).

# Survey Instruments Supplementary on-line material.
