# Supplementary material for: Training the next generation of physician researchers – Vanderbilt Medical Scholars Program
Source: BMC Med Educ. 2018 Jan 4;18:5. doi: 10.1186/s12909-017-1103-0 (PMC5753449; doi:10.1186/s12909-017-1103-0)
Supplement: Supplementary file 2 — Survey. (PDF 770 kb) [file 12909_2017_1103_MOESM2_ESM.pdf]

1. Introduction

Vanderbilt remains one of the few institutions that provides financial support for it's own medical students to complete a year of research. Your responses are important to us in being able to continue to secure program funding for future generations of Vanderbilt medical students.

To thank you for completing this survey, we will send you a $30 gift certificate to either Borders, Barnes and Noble, Starbucks, or Amazon.com (your choice). At the end of this survey, you will be automatically directed to a separate survey where you can enter your contact information and indicate the type of gift certificate you prefer. The two surveys are not linked, so your answers in this survey will remain ANONYMOUS.

The following survey consists of up to 66 questions and should take you less than 15 minutes to complete. Your survey answers will not be shared with anyone outside the office of the director of the Medical Scholars Program. Upon request, we may report ANONYMOUS summary data to program administrators (e.g. “70% of scholars presented their research at a national or international meeting"), but individual answers will not be shared.

2. Demographic Information

Questions on this page ask about specific demographic characteristics.

Upon request, we may report anonymous summary data to program administrators (e.g. "50% of all individuals that completed the Medical Scholars Program were female"), but individual answers will not be shared.

No attempt will be made to use answers on this page to identify specific individuals.

* 1. What is your date of birth?

	MM	DD	YYYY
Date of Birth		/	/

* 2. What is your gender?

( ) Female ( ) Male

* 3. What is your race? (check all that apply)

- [ ] Asian
- [ ] Black or African American
- [ ] Hispanic or Latino/a
- [ ] Native American
- [ ] Native Hawaiian or other Pacific Islander
- [ ] White or Caucasian
- [ ] Other (please specify)

* 4. Did you complete the Medical Scholars Program?

( ) Yes ( ) No

* 5. Would you recommend the Medical Scholars program to future medical students interested in research?

( ) Yes ( ) No

* 6. Why would you (or would you not) recommend the Vanderbilt Medical Scholars Program to future medical students interested in research?

In your answer, please let us know the following:
a) What attracted you to the Medical Scholars Program?
b) Did you find any specific features of the program beneficial?

* 7. What was your overall undergraduate GPA?

* 8. What was your total score on the MCAT?

* 9. Were you inducted into the AOA honor society during medical school?

- Yes
- No

* 10. Were you inducted into the AOA honor society after medical school?

- Yes
- No

3. Medical Scholars Program Completion

* 11. What year did you complete the Medical Scholars Program?

4. Medical School Completion

* 12. Have you completed medical school?

◯ Yes ◯ No

5. Medical School Completion: part 2

* 13. What year did you complete medical school?

6. Continuing Education

Questions in this section ask about your continuing education experiences after you completed medical school.

* 14. Which of the following best describes the phase of training you began immediately after graduation from medical school?

- None, I have not yet completed medical school.
- Clinical Internship
- Clinical Residency: traditional
- Clinical or Residency: fast-track
- Post-doctoral Research Fellowship
- Other type of training (please specify)

* 15. Please choose the description below which BEST describes yourCURRENT type of post-graduate CLINICAL TRAINING:
This refers to all internship, residency, fellowship, and CLINICAL degree programs after you completed medical school.

Please EXCLUDE any NON-CLINICAL training (such as a postdoctoral research fellowship). Questions about non-clinical training experiences will be asked later in this survey.

- I have neither started **NOR** ever had **ANY** post-graduate CLINICAL training.
- **Intern**
- **Resident** (with or without a concurrent CLINICAL degree program (e.g. MPH, MSCI))
- **Chief Resident** (with or without a concurrent CLINICAL degree program (e.g. MPH, MSCI))
- **Clinical Fellowship** (with or without a concurrent CLINICAL degree program (e.g. MPH, MSCI))
- My **ONLY CURRENT** post-graduate CLINICAL training is a postgraduate CLINICAL degree program (e.g. MPH, MSCI).
- I have completed all of my post-graduate CLINICAL training. (This refers to all internship, residency, fellowship, and post-graduate degree programs.)
- Other type of CLINICAL training (please specify)

7. Date of Post-Graduate Training Completion

* 16. When did you complete your training?

	MM		DD		YYYY
Date		/		/	

8. Continuing Education: Internship and Residency

* 17. In total, how many years have you completed in BOTH internship AND residency (combined)?

- None **(I have not started an internship or residency)**
- between one and eleven MONTHS
- 1 year
- 2 years
- 3 years
- 4 years
- 5 years
- 6 years
- 7 years
- 8 years
- 9 years
- 10 years

* 18. What was your INTERNSHIP experience? (Select all that apply.)

- None **(I did not do an internship.)**
- Family Practice
- Emergency Medicine
- General Surgery
- Internal Medicine
- Med-Peds
- Obstetrics and Gynecology
- Pathology
- Pediatrics
- Psychiatry
- Radiology
- Surgery (Other - includes ENT, orthopedics, plastic surgery, etc...)
- Other (please specify)

* 19. What was your FIRST type of residency experience? (Select all that apply.)

- None (**I did not do a residency.)**
- Family Practice
- Emergency Medicine
- General Surgery
- Internal Medicine
- Med-Peds
- Obstetrics and Gynecology
- Pathology
- Pediatrics
- Psychiatry
- Radiology
- Surgery (Other - includes ENT, orthopedics, plastic surgery, etc...)
- Other (please specify)

* 20. What was your SECOND type of residency experience? (Select all that apply.)

- [ ] None **(I did not do a second residency.)**
- [ ] Family Practice
- [ ] Emergency Medicine
- [ ] General Surgery
- [ ] Internal Medicine
- [ ] Med-Peds
- [ ] Obstetrics and Gynecology
- [ ] Pathology
- [ ] Pediatrics
- [ ] Psychiatry
- [ ] Radiology
- [ ] Surgery (Other - includes ENT, orthopedics, plastic surgery, etc...)
- [ ] Other (please specify)

* 21. Are you currently, or have you ever served as a chief resident?

- ( ) Yes
- ( ) No

* 22. Have you completed your fellowship training?

- ( ) **Yes**, I have completed my fellowship training.
- ( ) **No**, but I am currently in a fellowship.
- ( ) **No**, but I DO plan on doing a fellowship.
- ( ) **No,** and I DO NOT plan on doing a fellowship.

9. Continuing Education: Fellowship

* 23. Select your FIRST fellowship experience:

- None
- Adolescent Medicine
- Allergy and Immunology
- Behavioral Medicine
- Cardiology
- Clinical Immnology
- Clinical Pharmacology
- Clinical Scholar (i.e. RWJ)
- Consultative Psychiatry and Somatization
- Critical Care
- Dermatology
- Emergency Medicine (Adult)
- Emergency Medicine (Peds)
- Emergency Medicine (General)
- Endocrinology
- Epidemiology
- General Pediatrics
- Geriatric Rheumatology
- General Geriatrics
- GI
- GIM
- Health Services Research
- Hematology and/or Oncology
- HIV / AIDS
- Infectious Disease
- Medical Ethics
- Medical Genetics
- Nephrology
- Nutrition
- OB/GYN
- Occup and Environmental Medicine
- Oncology
- Pediatric Oncology
- Postdoctoral Research Fellow
- Preventive Medicine
- Primary Care
- Psychiatry
- Pulmonary Medicine
- Pulmonary / Critical Care
- Rheumatology
- Sports Medicine
- Substance Abuse
- Surgical Fellowship
- Other type of fellowship (please specify)

* 24. Select your SECOND fellowship experience:

- None
- Adolescent Medicine
- Allergy and Immunology
- Behavioral Medicine
- Cardiology
- Clinical Immnology
- Clinical Pharmacology
- Clinical Scholar (i.e. RWJ)
- Consultative Psychiatry and Somatization
- Critical Care
- Dermatology
- Emergency Medicine (Adult)
- Emergency Medicine (Peds)
- Emergency Medicine (General)
- Endocrinology
- Epidemiology
- General Pediatrics
- Geriatric Rheumatology
- General Geriatrics
- GI
- GIM
- Health Services Research
- Hematology and/or Oncology
- HIV / AIDS
- Infectious Disease
- Medical Ethics
- Medical Genetics
- Nephrology
- Nutrition
- OB/GYN
- Occup and Environmental Medicine
- Oncology
- Pediatric Oncology
- Postdoctoral Research Fellow
- Preventive Medicine
- Primary Care
- Psychiatry
- Pulmonary Medicine
- Pulmonary / Critical Care
- Rheumatology
- Sports Medicine
- Substance Abuse
- Surgical Fellowship
- Other type of fellowship (please specify)

* 25. Select your THIRD fellowship experience:

- None
- Adolescent Medicine
- Allergy and Immunology
- Behavioral Medicine
- Cardiology
- Clinical Immnology
- Clinical Pharmacology
- Clinical Scholar (i.e. RWJ)
- Consultative Psychiatry and Somatization
- Critical Care
- Dermatology
- Emergency Medicine (Adult)
- Emergency Medicine (Peds)
- Emergency Medicine (General)
- Endocrinology
- Epidemiology
- General Pediatrics
- Geriatric Rheumatology
- General Geriatrics
- GI
- GIM
- Health Services Research
- Hematology and/or Oncology
- HIV / AIDS
- Infectious Disease
- Medical Ethics
- Medical Genetics
- Nephrology
- Nutrition
- OB/GYN
- Occup and Environmental Medicine
- Oncology
- Pediatric Oncology
- Postdoctoral Research Fellow
- Preventive Medicine
- Primary Care
- Psychiatry
- Pulmonary Medicine
- Pulmonary / Critical Care
- Rheumatology
- Sports Medicine
- Substance Abuse
- Surgical Fellowship
- Other type of fellowship (please specify)

10. Continuing Education: Plans for Residency and Fellowship

The questions on this page ask about your future continuing education plans.
*****We understand that plans can change, so please provide your best guess in answering the questions on this page.**

* 26. Do you plan to complete a residency?

○ Yes ○ No

* 27. Do you plan to complete a fellowship?

○ Yes ○ No

11. Concurrent Degrees

* 28. Did you complete any additional, concurrent degrees (not including your M.D.) WHILE enrolled in medical school?

◯ Yes ◯ No

12. Completed Concurrent Degrees

* 29. Please select ALL additional, concurrent degrees that you earned WHILE YOU WERE ENROLLED IN MEDICAL SCHOOL (NOT including your M.D.).

- None
- DO
- EdD
- Dr. PH
- JD
- MA or MS
- MBA
- MEd
- MHS (Master of Health Sciences) or equivalent
- MPH (Master of Public Health) or equivalent
- MSCI (Master of Science in Clinical Investigation) or equivalent
- MSW
- PhD
- ScD
- Other degree (please specify)

13. Post-Graduate Degrees

* 30. Have you completed any post-graduate degrees following your MD?

◯ Yes ◯ No

14. Completed Post-Graduate Degrees

* 31. Please select the FIRST post-graduate degree (following your MD) you have earned.

- None
- DO
- EdD
- Dr. Epidemiology
- Dr. Public Health
- JD
- MA or MS
- MBA
- Other degree (please specify)
- MEd
- MHS (Master of Health Sciences) or equivalent
- MPH (Master of Public Health) or equivalent
- MSCI (Master of Science in Clinical Investigation) or equivalent
- MSW
- PhD
- ScD

* 32. Please select the SECOND post-graduate degree (following your MD) you have earned.

- None
- DO
- EdD
- Dr. Epidemiology
- Dr. Public Health
- JD
- MA or MS
- MBA
- Other degree (please specify)
- MEd
- MHS (Master of Health Sciences) or equivalent
- MPH (Master of Public Health) or equivalent
- MSCI (Master of Science in Clinical Investigation) or equivalent
- MSW
- PhD
- ScD

15. Formal Training Experiences

The questions on this page ask about formal training experiences, which include structured courses, seminars, or workshops.

33. Indicate the following areas in which you have ever received formal training.
(Choose all that apply)

- [ ] Teaching
- [ ] Curriculum / Program Development / Evaluation
- [ ] Clinical Research
- [ ] Laboratory Research
- [ ] Administration of Educational Programs, Curricula, Fellowships, or Courses
- [ ] Clinical / Business / Research Administration
- [ ] Writing Manuscripts for Publication
- [ ] Presenting your work
- [ ] Grant Writing / Obtaining External Funding
- [ ] Working in Groups (group process, group dynamics)
- [ ] Time Management
- [ ] Leadership
- [ ] Communication Across Cultural Differences
- [ ] Communication with Patients

* 34. In which of the following areas do you think formal training would be valuable for future Medical Scholars Program participants?
(Choose all that apply)

- [ ] Teaching
- [ ] Curriculum / Program Development / Evaluation
- [ ] Clinical Research
- [ ] Laboratory Research
- [ ] Administration of Educational Programs, Curricula, Fellowships, or Courses
- [ ] Clinical / Business / Research Administration
- [ ] Writing Manuscripts for Publication
- [ ] Presenting your work
- [ ] Grant Writing / Obtaining External Funding
- [ ] Working in Groups (group process, group dynamics)
- [ ] Time Management
- [ ] Leadership
- [ ] Communication Across Cultural Differences
- [ ] Communication with Patients

16. Current Post-Graduate Education

* 35. Are you currently enrolled in a degree-granting program?

◯ Yes ◯ No

17. Current Degree-Granting Program

* 36. Please the type of degree-granting program in which you areCURRENTLY ENROLLED.
(Choose all that apply.)

- [ ] None
- [ ] DO
- [ ] Dr. Epidemiology
- [ ] Dr. Public Health
- [ ] EdD
- [ ] JD
- [ ] MA or MS
- [ ] MBA
- [ ] MD
- [ ] MEd
- [ ] MHS (Master of Health Sciences) or equivalent
- [ ] MPH (Master of Public Health) or equivalent
- [ ] MSCI (Master of Science in Clinical Investigation) or equivalent
- [ ] MSW
- [ ] PhD
- [ ] ScD
- [ ] Other degree (please specify)

18. Current Position

The questions in the remaining sections ask about your current position.

* 37. On average, how many hours do you currently work per week?
(This includes all time spent in class, clinic, lab, or at the office.)

* 38. In which of the following areas do you spend themajority of your time at work?
(Choose all that apply.)

- [ ] Patient Care
- [ ] Teaching
- [ ] Curriculum / Program Development and / or Evaluation
- [ ] Research: Publication preparation, Presentations, Grant Writing / Obtaining Funding
- [ ] Research: Performing Studies, Data Analysis
- [ ] Administration: Clinical / Business / Research (non-Educational)
- [ ] Administration of Educational Curriculum, Program, Rotation, Fellowship, or Course
- [ ] Committee Work
- [ ] Community Outreach / Advocacy / Education
- [ ] Your OWN Continuing Education Activities (including coursework)
- [ ] Other Type of Activity (please specify)

* 39. Estimate the percent of your time working in each of the following areas.
If you do not spend any time in a particular area, please indicate this by clicking on "0%" for that specific area.
(The sum of all percents in this section should equal 100%)

	0%	10%	20%	30%	40%	50%	60%	70%	80%	90%	100%
Patient Care	○	○	○	○	○	○	○	○	○	○	○
Teaching	○	○	○	○	○	○	○	○	○	○	○
Curriculum / Program Development and / or Evaluation	○	○	○	○	○	○	○	○	○	○	○
Research: Publication preparation, Presentations, Grant Writing / Obtaining Funding	○	○	○	○	○	○	○	○	○	○	○
Research: Performing Studies, Data Analysis	○	○	○	○	○	○	○	○	○	○	○
Administration: Clinical / Business / Research (non-Educational)	○	○	○	○	○	○	○	○	○	○	○
Administration of Educational Curriculum, Program, Rotation, Fellowship, or Course	○	○	○	○	○	○	○	○	○	○	○
Committee Work	○	○	○	○	○	○	○	○	○	○	○
Community Outreach / Advocacy / Education	○	○	○	○	○	○	○	○	○	○	○
Your OWN Continuing Education Activities (including coursework)	○	○	○	○	○	○	○	○	○	○	○
Other type of Activity	○	○	○	○	○	○	○	○	○	○	○

* 40. What about the Medical Scholars Program has helped you most in your current career stage?

* 41. Rate the level of support (of all kinds) that you feel you receive from your present working environment for the following activities:

	Not Applicable	No Support	A Little Support	A Moderate Amount of Support	A Lot of Support
Patient Care	○	○	○	○	○
Teaching	○	○	○	○	○
Research	○	○	○	○	○
Administration	○	○	○	○	○
Preparing for Publications or Outside Presentations	○	○	○	○	○
Grant Writing / Obtaining External Funding	○	○	○	○	○
Committee Work	○	○	○	○	○
Your OWN Continuing Education Activities (including coursework)	○	○	○	○	○

* 42. Do you currently have an academic appointment, defined as either a faculty position (clinical or research) or administrative position at a university or medical center?

◯ Yes ◯ No

19. Academic Appointment

* 43. What type of academic appointment do you currently hold?

- ◯ Administrator
- ◯ Adjunct Appointment
- ◯ Clinician (Private Practice)
- ◯ Clinical Instructor
- ◯ Instructor
- ◯ Assistant Professor
- ◯ Associate Professor
- ◯ Professor

* 44. Which of the following describes your current academic position?

- ◯ Clinical Track
- ◯ non-Tenure Track
- ◯ Tenure Track
- ◯ Other type of academic position (please specify)

* 45. Please indicate the title(s) of any administrative position(s) that you currently fill. (Choose all that apply)

- ☐ None
- ☐ Clerkship Director
- ☐ Clinical Rotation Director
- ☐ Program Director
- ☐ Associate Program Director
- ☐ Course Director
- ☐ Chair Academic Affairs Committee
- ☐ Division Director
- ☐ Department Chair
- ☐ Assistant Division or Department Director
- ☐ Chief Resident / ACS
- ☐ Clinical Director of a Commercial Clinic / HMO / Hosp Clinic
- ☐ Principal / Co-principal Investigator
- ☐ Dean / Assistant Dean
- ☐ Director, Office of Student Research
- ☐ Fellowship Officer
- ☐ Other (please specify)

20. External Funding

* 46. Currently, what percent of your salary is supported through grants or other external funding?

- None
- less than 25%
- 25%-49%
- 50%-74%
- 75% or more
- I don't know

* 47. Have you ever written OR participated in writing a grant to obtain funding for all or part of your salary and/or for a project?

- Yes
- No

21. External Funding: part 2

The questions on this page ask about the types of grant applications that you have either submitted (as the Principal Investigator) OR only participated in writing.

* 48. Please indicate which of the following type(s) grant applications that you have EVER SUBMITTED as Principal Investigator. (Choose all that apply)

- [ ] None
- [ ] Career Development Award from a non-NIH governmental agency (e.g. DoD, CDC, VA)
- [ ] Career Development Award from a Private Foundation (e.g. Doris Duke, Burroughs-Wellcome)
- [ ] Career Development Award from an industry source (e.g. Pfizer)
- [ ] NIH Individual Postdoctoral Research Fellowship (F32)
- [ ] NIH Individual Career Development Award (K08, K23, or other K-series award)
- [ ] NIH Director's Pioneer Award (DP1)
- [ ] NIH Director's New Innovator Program (DP2)
- [ ] NIH Loan Repayment Program (L series)
- [ ] NIH Pathway to Independence Award (K99/R00)
- [ ] NIH Research Project Grant (R01 or other R-series)
- [ ] NIH Research Program Project / Center Awards (P-series)
- [ ] Other NIH grant
- [ ] Research Grant from a non-NIH governmental agency
- [ ] Research Grant from a Private Foundation (e.g. Doris Duke, Burroughs-Wellcome)
- [ ] Industry funding for clinical research or clinical trials (e.g. Pfizer)
- [ ] Other type of grant not listed above (please specify)

* 49. Please indicate which of the following type(s) grant applications that you have EVER PARTICIPATED IN WRITING, but YOU WERE NOT the Principal Investigator.
(Choose all that apply)

- [ ] None
- [ ] Career Development Award from a non-NIH governmental agency (e.g. DoD, CDC, VA)
- [ ] Career Development Award from a Private Foundation (e.g. Doris Duke, Burroughs-Wellcome)
- [ ] Career Development Award from an industry source (e.g. Pfizer)
- [ ] NIH Individual Postdoctoral Research Fellowship (F32)
- [ ] NIH Individual Career Development Award (K08, K23, or other K-series award)
- [ ] NIH Director's Pioneer Award (DP1)
- [ ] NIH Director's New Innovator Program (DP2)
- [ ] NIH Loan Repayment Program (L series)
- [ ] NIH Pathway to Independence Award (K99/R00)
- [ ] NIH Research Project Grant (R01 or other R-series)
- [ ] NIH Research Program Project / Center Awards (P-series)
- [ ] Other NIH grant
- [ ] Research Grant from a non-NIH governmental agency
- [ ] Research Grant from a Private Foundation (e.g. Doris Duke, Burroughs-Wellcome)
- [ ] Industry funding for clinical research or clinical trials (e.g. Pfizer)
- [ ] Other type of grant not listed above (please specify)

22. External Funding: part 3

The questions on this page ask about current and previous grant funding that you have been awarded as Principal Investigator.

* 50. Please indicate which of the following type(s) of CURRENT external grant funding on which you NOW SERVE as PRINCIPAL INVESTIGATOR.
(Choose all that apply)

- [ ] None
- [ ] Career Development Award from a non-NIH governmental agency (e.g. DoD, CDC, VA)
- [ ] Career Development Award from a Private Foundation (e.g. Doris Duke, Burroughs-Wellcome)
- [ ] Career Development Award from an industry source (e.g. Pfizer)
- [ ] NIH Individual Postdoctoral Research Fellowship (F32)
- [ ] NIH Individual Career Development Award (K08, K23, or other K-series award)
- [ ] NIH Director's Pioneer Award (DP1)
- [ ] NIH Director's New Innovator Program (DP2)
- [ ] NIH Loan Repayment Program (L series)
- [ ] NIH Pathway to Independence Award (K99/R00)
- [ ] NIH Research Project Grant (R01 or other R-series)
- [ ] NIH Research Program Project / Center Awards (P-series)
- [ ] Other NIH grant
- [ ] Research Grant from a non-NIH governmental agency
- [ ] Research Grant from a Private Foundation (e.g. Doris Duke, Burroughs-Wellcome)
- [ ] Industry funding for clinical research or clinical trials (e.g. Pfizer)
- [ ] Other type of grant not listed above (please specify)

* 51. Please indicate which of the following type(s) of PREVIOUS external grant funding you have EVER BEEN AWARDED as PRINCIPAL INVESTIGATOR.
This DOES NOT include CURRENT grant funding.
(Choose all that apply)

- [ ] None
- [ ] Career Development Award from a non-NIH governmental agency (e.g. DoD, CDC, VA)
- [ ] Career Development Award from a Private Foundation (e.g. Doris Duke, Burroughs-Wellcome)
- [ ] Career Development Award from an industry source (e.g. Pfizer)
- [ ] NIH Individual Postdoctoral Research Fellowship (F32)
- [ ] NIH Individual Career Development Award (K08, K23, or other K-series award)
- [ ] NIH Director's Pioneer Award (DP1)
- [ ] NIH Director's New Innovator Program (DP2)
- [ ] NIH Loan Repayment Program (L series)
- [ ] NIH Pathway to Independence Award (K99/R00)
- [ ] NIH Research Project Grant (R01 or other R-series)
- [ ] NIH Research Program Project / Center Awards (P-series)
- [ ] Other NIH grant
- [ ] Research Grant from a non-NIH governmental agency
- [ ] Research Grant from a Private Foundation (e.g. Doris Duke, Burroughs-Wellcome)
- [ ] Industry funding for clinical research or clinical trials (e.g. Pfizer)
- [ ] Other type of grant not listed above (please specify)

23. Professional Development

* 52. Indicate which of the following types of publications that you have EVER authored or co-authored. (Choose all that apply)

- [ ] None
- [ ] Abstracts (for National or International Conference or Meeting)
- [ ] Medical Book or Book Chapter
- [ ] Medical Review
- [ ] Medical Manuscript / Original Research
- [ ] Letter to the Editor
- [ ] Book Review
- [ ] Other type of publication (please specify)

* 53. As of today's date, what is the TOTAL number of each of the following publications and presentations have you had SINCE YOU STARTED the Medical Scholars Program? (Count work that is "in press" but not work that is "submitted", or "in preparation.")

	0	1	2	3	4	5	6	7	8	9	10 or more
Peer Reviewed Manuscripts **(First Author)**	◯	◯	◯	◯	◯	◯	◯	◯	◯	◯	◯
Peer Reviewed Manuscripts **(Not First Author)**	◯	◯	◯	◯	◯	◯	◯	◯	◯	◯	◯
Completed Abstracts	◯	◯	◯	◯	◯	◯	◯	◯	◯	◯	◯
Paper presentations at **regional** academic or continuing education meetings	◯	◯	◯	◯	◯	◯	◯	◯	◯	◯	◯
Paper presentations at **national / international** academic or continuing education meetings	◯	◯	◯	◯	◯	◯	◯	◯	◯	◯	◯

* 54. How many of each of the following directly arose from your year of research in the Medical Scholars Program?

	0	1	2	3	4	5 or more
Peer Reviewed Manuscripts **(First Author)**	○	○	○	○	○	○
Peer Reviewed Manuscripts **(Not First Author)**	○	○	○	○	○	○
Completed Abstracts	○	○	○	○	○	○
Paper presentations at **regional** academic or continuing education meetings	○	○	○	○	○	○
Paper presentations at **national/international** academic or continuing education meetings	○	○	○	○	○	○

* 55. How many of each of the following directly arose from your specific research project in the Medical Scholars Program?

	0	1	2	3	4	5 or more
Peer Reviewed Manuscripts **(First Author)**	○	○	○	○	○	○
Peer Reviewed Manuscripts **(Not First Author)**	○	○	○	○	○	○
Completed Abstracts	○	○	○	○	○	○
Paper presentations at **regional** academic or continuing education meetings	○	○	○	○	○	○
Paper presentations at **national/international** academic or continuing education meetings	○	○	○	○	○	○

24. Career Goals

The questions on this page ask about your long-term career goals.
We understand that plans can change, so please indicate your best guess at this time.

* 56. What was your PRIMARY LONG-TERM career goal when you ENTERED the Medical Scholars Program ?

- Undecided
- Full-Time academic faculty: RESEARCH AND TEACHING
- Full-Time academic faculty: RESEARCH AND CLINICAL CARE
- Full-Time academic faculty: TEACHING AND CLINICAL CARE
- Non-university research scientist / clinician
- Full-time non-academic clinician
- Position in Government Agency (state or federal)
- Medical or Healthcare Administration
- Other (please specify)

* 57. What is your CURRENT PRIMARY LONG-TERM career goal?

- Undecided
- Full-Time academic faculty: RESEARCH AND TEACHING
- Full-Time academic faculty: RESEARCH AND CLINICAL CARE
- Full-Time academic faculty: TEACHING AND CLINICAL CARE
- Non-university research scientist / clinician
- Full-time non-academic clinician
- Position in Government Agency (state or federal)
- Medical or Healthcare Administration
- Other (please specify)

* 58. Has your primary long-term career goal changed as a result of your participation in the Medical Scholars Program?

Yes No

* 59. Please let us know HOW and WHY the Medical Scholars Program may have changed (or confirmed) your original primary career goal.

* 60. Which PRIMARY AREA would you like to focus on in your long-term career?

- ○ Basic Science Research
- ○ Clinical or Translational Research
- ○ Clinical Care
- ○ Global Health
- ○ Healthcare Policy
- ○ Medical Administration
- ○ Public Health / Epidemiology
- ○ Other (please specify)

* 61. How extensively do you plan to be involved with research during your medical career?

- ○ Exclusively
- ○ Significantly involved
- ○ Somewhat involved
- ○ Involved in a limited way
- ○ Not involved

25. Student Debt

* 62. Estimate the amount of ACADEMIC Debt (if any) that you currently have (i.e., student loans, loans for tuition/fees/living expenses while in college, medical school, and graduate school).

- $0
- $1-24,999
- $25,000-49,999
- $50,000-74,999
- $75,000-99,999
- $100,000-124,999
- $125,000-149,999
- $150,000-174,999
- $175,000-199,999
- $200,000 or more

* 63. Estimate the amount of HOUSEHOLD Debt (if any) that you currently have (i.e.,home mortgage, car loan, medical expenses, etc...).

- $0
- $1-24,999
- $25,000-49,999
- $50,000-74,999
- $75,000-99,999
- $100,000-124,999
- $125,000-149,999
- $150,000-174,999
- $175,000-199,999
- $200,000 or more

* 64. Estimate the amount of OTHER Debt (if any) that you currently have (i.e.,credit cards).

- $0
- $1-24,999
- $25,000-49,999
- $50,000-74,999
- $75,000-99,999
- $100,000-124,999
- $125,000-149,999
- $150,000-174,999
- $175,000-199,999
- $200,000 or more

* 65. What is the highest education level attained by your parents or guardians?

	Mother (or Female guardian)	Father (or Male guardian)
Less than high school education	☐	☐
High school education	☐	☐
Some college	☐	☐
Associate's degree	☐	☐
Bachelor's degree	☐	☐
Master's degree	☐	☐
Professional degree (JD, MD, DDS, DVM, etc)	☐	☐
PhD	☐	☐
Not applicable	☐	☐
Prefer not to answer	☐	☐

26. Additional feedback

* 66. Please take this opportunity to give us additional feedback on the Medical Scholars Program in the box below:

Thank you for completing the Medical Scholars Program Alumni survey.

Please press the "Done" button below, and you will be directed automatically to a separate survey where you can enter your contact information and indicate the type of gift certificate you prefer. The two surveys ARE NOT linked, so your answers in this survey will remain anonymous.

Medical Scholars Alumni Contact Information & Gift Card Selection v. 2

1. Contact Information

Thank you for completing the previous survey. Your answers on the previous survey<u>ARE NOT</u> linked to your answers below, so all of your previous responses will remain anonymous.

The following survey questions only ask for your contact information and gift card choice.

* 1. Please provide your current contact information below. (This is how we will contact you to send your gift card.)

Your Name:

Home Address:

Apartment #:

City/Town:

State: -- select state --

ZIP/Postal Code:

Country:

Personal (Permanent) Email Address:

Phone Number:

* 2. What is your current work address?
(Please enter your school address and location if you are currently a student.)

Current Employer:

Work Address:

Address 2:

City/Town:

State: -- select state --

ZIP/Postal Code:

Country:

Work Email Address:

Phone Number:

* 3. Please provide the name and contact information of a person who is likely to know where you can be reached in the future so that we can follow up with your career progress.

Contact Name:

Relationship to you:
(parent, other family member, etc...)

Street Address:

Apartment #:

City/Town:

State: -- select state --

ZIP/Postal Code:

Country:

Email Address:

Phone Number:

* 4. Are you planning to move in the next 12 months?

○ Yes ○ No ○ I don't know

5. If you plan to move sometime in the next 12 months, please complete as much of the following information that you know.

Future Employer

Future Home Street Address:

Apartment #:

City/Town:

State: -- select state --

ZIP/Postal Code:

Country:

Phone Number:

Please press the "Next" button below to choose the gift card you prefer.

2. Gift Card

6. Which type of gift card do you prefer?

- ○ Starbucks
- ○ Amazon.com
- ○ Borders
- ○ Barnes and Noble
